# Circ-PNPT1 contributes to gestational diabetes mellitus (GDM) by regulating the function of trophoblast cells through miR-889-3p/PAK1 axis

**DOI:** 10.1186/s13098-021-00678-9

**Published:** 2021-06-01

**Authors:** Li Zhang, Ming Zeng, Fei Tang, Jun Chen, Dongmei Cao, Ze-nan Tang

**Affiliations:** 1 Department of Obsterics, Maternal and Child Health Hospital of Hubei Province, No.745 Wulu Road, Hongshan District, Wuhan City, 430070 Hubei Province China; 2 Department of Public Course, Hubei Communication Technical College, Wuhan City, 430079 Hubei China

**Keywords:** Circ-PNPT1, miR-889-3p, PAK1, Gestational diabetes mellitus, Exosomes

## Abstract

**Background:**

Gestational diabetes mellitus (GDM) is the most common medical complication of pregnancy. CircRNA polyribonucleotide nucleotidyltransferase 1 (circ-PNPT1) has been found to be abnormally expressed in GDM patients. However, function and mechanism of circ-PNPT1 in GDM remain largely undefined.

**Methods:**

Levels of circ-PNPT1, microRNA (miR)-889-3p and PAK1 (p21 (RAC1) activated kinase 1) were detected using quantitative real-time polymerase chain reaction and Western blot assays. Cell viability, apoptosis, migration and invasion were determined using cell counting kit-8 assay, flow cytometry, transwell and wound healing assays, respectively. The binding interaction between miR-889-3p and circ-PNPT1 or PAK1 was verified using dual-luciferase reporter, RNA immunoprecipitation (RIP) and RNA pull-down assays. Exosomes were obtained from culture media by the use of commercial kits and qualified by transmission electron microscopy (TEM).

**Results:**

Circ-PNPT1 was highly expressed in the placental tissues of GDM and high glucose (HG)-induced trophoblast cells. Knockdown of circ-PNPT1 reversed HG-induced arrest of trophoblast cell viability, migration, invasion and the promotion of cell apoptosis. Mechanistically, we confirmed circ-PNPT1 could promote the expression of PAK1, the target of miR-889-3p, by directly sponging miR-889-3p, and circ-PNPT1 regulated HG-induced trophoblast cell dysfunction by miR-889-3p/PAK1 axis. Further studies showed circ-PNPT1 was packaged into exosomes and could be internalized by surrounding trophoblast cells.

**Conclusion:**

Circ-PNPT1 promoted HG-induced trophoblast cell biological dysfunction through miR-889-3p/PAK1 axis. Meanwhile, it could be transferred from HG-induced trophoblast cells to surrounding untreated cells via exosomes.

**Supplementary Information:**

The online version contains supplementary material available at 10.1186/s13098-021-00678-9.

## Introduction

Gestational diabetes mellitus (GDM), referring to glucose intolerance that is initiated during pregnancy, is the most common medical complication of pregnancy [1]. According to the International Diabetes Federation (IDF), GDM occurs in ~ 14% globally and 21% in Asia in 2017 [2]. In China, the incidence of GDM was reported to be 11.91%, much higher than Japan, Korea, and Thailand with a GDM prevalence of less than 8.0% [3]. GDM is highlighted by hyperglycemia and disorder of carbohydrate metabolism, aside from the immediate perinatal risk, GDM increases the risk of metabolic diseases in mothers and children [4, 5]. It is one of the main cause of maternal and neonatal adverse outcomes. The placenta is a crucial organ for nutrition exchange, gas exchange and blood circulation between mother and fetus during pregnancy [6]. Besides that, placenta has significant endocrine functions, hormones secreted by placenta antagonize insulin and enhance insulin resistance, which in turn cause GDM [7, 8]. The abnormal placental function is involved in GDM-related adverse pregnancy outcomes [9, 10]. Additionally, trophoblast cells with normal biological function are critical for placenta development, thus, targeting HG-induced trophoblast cell dysfunction may be an effective strategies for investigating the pathogenesis of GDM.

Circular RNAs (circRNAs) are a class of non-coding RNA molecules highlighted by covalently closed circles that lack the 3′ and 5′ ends, so they can resistant to RNase R [11]. Additionally, circRNAs are widely in mammal cells, and show high stability and specie-, tissue-, cell- or disease-specific expression pattern [12]. Besides that, growing evidence has suggested that circRNAs have critical roles in regulating diverse biological processes, including cell growth, metastasis and epithelial–mesenchymal transition [13–15]. These characters make circRNAs to be one of ideal biomarkers for future therapeutic interventions [16]. Recently, aberrant expression of circRNAs has been revealed to be associated with the pathological conditions of various diseases, including GDM [17]. CircRNA polyribonucleotide nucleotidyltransferase 1 (circ-PNPT1, ID: hsa_circ_0054633) is a novel identified functional circRNA, which is derived from the PNPT1 gene and locates at chr2:55861197-55913579. It was found to be differentially expressed in GDM patients and might be a risk factor in GDM development [18]. However, the action and specific regulatory mechanism of circ-PNPT1 in GDM are still not fully elucidated.

CircRNAs have been observed in secreted exosomes, the membrane-bound vesicles 40–150 nm in diameter [19]. Exosomes are critical mediators of cell to cell communication, both locally and systemically; they deliver cargo that may include proteins, RNAs and lipids to neighboring or distant cells, thereby modulating the biological processes of recipient cells [20, 21]. Recently, it has been revealed that circRNAs are abundant and stable in exosomes, and exosomal circRNAs have emerged to play important roles in cancer diagnosis and progression [22, 23]. However, investigations on the biological functions of exosomal circ-PNPT1 in GDM are largely undefined.

In this review, high glucose (HG)-induced trophoblast cells were used to mimic type 2 diabetes mellitus (T2dM) condition in vitro. Then the pathological role and potential regulatory network of circ-PNPT1 in the dysfunction of trophoblast cells were investigated. Besides that, we also attempted to explore whether circ-PNPT1 performed its effects via exosomes.

## Materials and methods

### Collection of tissue samples

A total of 19 parturient women diagnosed with GDM and 19 parturient healthy control women from the Maternal and Child Health Hospital of Hubei Province were included in this study. GDM was diagnosed according to the criteria of the International Association of the Diabetes and Pregnancy Study Groups (IADPSG), and women with pre-gestational diabetes; twins (multiple) pregnancy; pregnancy complications, such as preeclampsia; primary hypertension; and severe liver, kidney or heart diseases were excluded. At the time of delivery, placental biopsies from GDM women and healthy women were collected and instantly preserved at – 80 °C. This work was authorized by the Ethics Committee of the Maternal and Child Health Hospital of Hubei Province, and written informed consent was collected from all participants.

### Cell culture, transfection and treatment

Human placenta trophoblast cells HTR8/SVneo (Jining Cell Culture Center, Shanghai, China) were maintained in a 5% CO_2_ humidified atmosphere at 37 °C with the RPMI 1640 medium (Cat# 22400105, Thermo Fisher Scientific, Waltham, CA, USA) harboring 10% fetal bovine serum (FBS) (Cat# S9020, Solarbio, Shanghai, China) and 1% streptomycin-penicillin (Cat# P1400, Solarbio) [24].

The full-length cDNA of circ-PNPT1 was amplified and cloned into over expression vector pCD5-ciR (Geneseed, Guangzhou, China) to overexpress circ-PNPT1 (circ-PNPT1), and the mock vector with no circ-PNPT1 sequence was used as a control (pCD5-ciR). The specific siRNA targeting the back-splice junction site of circ-PNPT1 (si-circ-PNPT1) and a negative control siRNA (si-NC) were synthesized by Geneseed. The pcDNA-PAK1 overexpression vector (PAK1) and plasmid containing scrambled sequences (pcDNA), miRNA mimics (miR-889-3p), inhibitors (anti-miR-889-3p) and negative controls (miR-NC or anti-miR-NC) were synthesized by GenePharma (Shanghai, China). Until 70–80% of confluence, the transfections in HTR8/SVneo cells (2 × 10^5^ cells/well) were conducted using Lipofectamine 2000 according to the manufacturer's instructions (Invitrogen, Carlsbad, CA, USA).

HTR8/SVneo cells were subjected to glucose exposure (Cat# G8150, Solarbio) to mimic T2dM condition in vitro. Cells were cultured in the above medium containing 5 mmol/L glucose (normal glucose, control) or 25 mmol/L glucose (HG) for 48 h [25].

### Cell proliferation assay

After assigned transfection and/or treatment, HTR8/SVneo cells (3 × 10^3^ cells/well) were seeded in 96-well cell culture plates, then cell counting kit-8 (CCK-8) solution (Cat# C0037, Beyotime, Shanghai, China) (10 μL/well) was added into per well and incubated for 2 h. The absorbance at 450 nm was detected using a microplate reader to calculate cell viability [26].

### Cell apoptosis assay

HTR8/SVneo cells, following assigned transfection and/or treatment, were rinsed two times with 1 × PBS and then resuspended with 1 × buffer to make the density at 1 × 10^6^ cells/mL. After that, 100 μL cell suspension was gently mixed with Annexin V-fluorescein isothiocyanate (FITC) (Cat#560931, 5 μL) and propidium iodide (PI) (Cat#556463, 5 μL) (BD Biosciences, Franklin Lakes, NJ, USA) away from light at 37 °C. Finally, the FACSCanto II flow cytometry (BD Biosciences) was used to assess cell apoptosis [26].

### Cell migration and invasion assays

The transwell chambers (Costar, Cambridge, MA, USA) coated with or without Matrigel (500 ng/μL; BD Biosciences) were employed to analyze cell migratory and invasive capabilities. After assigned transfection and/or treatment, HTR8/SVneo cell suspension (1 × 10^5^ cells/mL or 5 × 10^5^ cells/mL in 150 μL serum-free medium for migration or invasion, respectively) was seeded on the top chamber, and lower chamber was filled with 600 μL RPMI 1640 medium with 10% FBS to use as the chemoattractant. 24 h later, the cells passing through the membrane were fixed with paraformaldehyde and stained with 0.1% crystal violet (Cat#C0121, Beyotime). Then the crystal violet-stained cells were imaged and counted (magnification 200 ×) [27].

Wound healing assay was also used to detect cell migration. After assigned transfection and/or treatment, ~ 2 × 10^6^ HTR8/SVneo cells in complete medium were seeded onto 6-well plates with the same number. Until 80–95% confluence, scratch wounds were made using a 200 μL sterile pipette tip. The plates were washed with 1 mL of fresh growth medium to remove the suspended cells and then cultured in the incubator at 37 °C. Images were captured at 0 and 24 h and wound widths were quantitatively examined using a standard caliper [27].

### Quantitative real-time polymerase chain reaction (qRT-PCR)

Total RNA was isolated from the cultured cells according to the instructions of the Trizol kit (Invitrogen). NanoDrop 8000 (Thermo Fisher Scientifc) was used to detect the concentration and purity of RNA. Then, 3 μg of RNA was reverse transcribed into cDNA using the PrimeScript RT reagent kit or PrimeScript miRNA cDNA Synthesis Kit (Takara, Kusatsu, Japan), and qRT-PCR was then conducted with the SYBR Green PCR Master Mix (Thermo Fisher Scientific). For the detection of circRNA and genes, the reaction for each sample was conducted at 95 °C for 3 min, followed by 40 cycles of 95 °C for 15 s and 60 °C for 1 min. For miRNA measurement, the reaction for each sample was performed at 94 °C for 2 min, followed by 40 cycles of 94 °C for 20 s, 60 °C for 30 s, and 72 °C for 30 s. U6 or glyceraldehyde 3-phosphate dehydrogenase (GAPDH) was regarded as the internal reference and the relative fold changes were calculated by the 2^−ΔΔCt^ method [28]. The primer sequences for qRT-PCR were listed:

circ-PNPT1: F 5′-AGCATGTTAGGCAATGTTGAT-3′, R 5′-TTTACTGACCGCTGTGACCA-3′;

PNPT1: F 5′-CCTTCCCAGTTTATGCCTTTGG-3′, R 5′-AAAGAGCGGTCTAATTGAACGAT-3′;

PAK1: F 5′-ATTCCCTTTTCCACGGAGCC-3′, R 5′-TCTGAGGCAGGAGGTGGTAA-3′;

GADPH: F 5′-CCCACATGGCCTCCAAGGAGTA-3′, R 5′-GTGTACATGGCAACTGTGAGGAGG-3′;

miR-889-3p: F 5′-ACACTCCAGCTGGGTTAATATCGGACAAC-3′, R 5′-TGGTGTCGTGGAGTCG-3′;

U6: F 5′-CTCGCTTCGGCAGCACA-3′, R 5′-AACGCTTCACGAATTTGCGT-3′.

### RNase R digestion

Approximately 3 μg of total RNAs were treated without or with 3 U/μg of RNase R (Cat# RNR07250, Epicenter, Madison, WI, USA) at room temperature for 20 min, followed by purification using RNeasy MinElute Cleanup Kit (Qiagen, Tokyo, Japan). At last, the abundances of PNPT1 mRNA and circ-PNPT1 were determined using qRT-PCR assay [27].

### Subcellular fractionation

Referring to the instructions of manufacturer, the cytosolic and nuclear fractions were isolated and collected employing the NE-PER™ Nuclear and Cytoplasmic Extraction Reagents Kit (Epicentre Technologies, Madison, WI, USA). Thereafter, the expression levels of circ-PNPT1 in nuclear and cytoplasm fractions of HTR8/SVneo cells were assayed using qRT-PCR. U6 was regarded as a nuclear biomarker, and GAPDH was regarded as a cytoplasm biomarker [27].

### Dual-luciferase reporter assay

The sequence of circ-PNPT1 and PAK1 3′UTR, harboring the miR-889-3p seed region or a mutant sequence, was cloned into the pmirGLO luciferase vectors (GeneCreat, Wuhan, China), named as WT-circ-PNPT1, MUT-circ-PNPT1, WT-PAK1 3′UTR, or MUT-PAK1 3′UTR, respectively. Then sub-confluent (70–80%) HTR8/SVneo cells were seeded into 48-well plates and co-transfected with these constructed luciferase reporter vectors together with miR-889-3p mimic or miR-NC. After 48 h of transfection, firefly activity was analyzed using the dual luciferase reporter system (GeneCreat) [27].

### RNA immunoprecipitation (RIP) assay

Anti-Ago2 or anti-IgG antibody was coupled to the protein A/G magnetic beads (Merck, Darmstadt, Germany) and then incubated with HTR8/SVneo cell lysate overnight at 4 °C. After removing the protein through the interaction with protease K buffer (Cat# AM2548, Invitrogen), the immunoprecipitated RNAs were eluted, and purified RNA was analyzed using qRT-PCR assay [29].

### RNA pull-down assay

Biotin-labeled miR-889-3p probe (bio-miR-889-3p) and miR-NC probe (bio-miR-NC) were synthesized by Geneseed. HTR8/SVneo cells were lysed in lysis buffer and then incubated with miR-889-3p-specific probes-streptavidin beads (Life Technologies, Carlsbad, CA, USA) mixture at 37 °C overnight. The beads were washed and the mixture was purified with TRIzol (Cat# 15596018, Invitrogen). Finally, the detection of circ-PNPT1 was conducted using qRT-PCR [29].

### Western blot

Protein samples isolated from cultured cells using the RIPA lysis buffer (Cat# P0013C, Beyotime) were electrophoresed on 10% sodium dodecyl sulphate–polyacrylamide gel electrophoresis and then transferred to polyvinylidene fluoride (PVDF) membranes (Millipore, Billerica, MA, USA). After blocking for 1 h with 5% skim milk, the membranes were incubated with specific primary antibodies at 4 °C overnight, followed by the secondary antibodies at 37 °C for 2 h. The immunoreactive signals were visualized by enhanced chemiluminescence reagent kit (Millipore). The primary antibodies used in this study: anti-PAK1 (1:1000, Cat#2602, Cell signaling, Boston, MA, USA), anti-CD63 (1:1000, ab134045, Abcam, Shanghai, China), anti-CD9 (1:2000, ab92726, Abcam), and anti-GAPDH (ab18602, 1: 5000, Abcam) [26].

### Exosome isolation

Culture medium was centrifuged at 3000*g* for 15 min, and filtered through a 0.22-µm PVDF filter (Millipore) to remove cells and cellular debris. Then the filtered culture medium was mixed with the Exoquick exosome precipitation solution (System Biosciences, CA, USA) at a ratio of 1:5 and refrigeration for at least 12 h. Thereafter, the mixture was re-centrifuged at 1500*g* for 30 min, the supernatant was discarded and exosomes were collected. Purified exosomes were resuspended in approximately 100 μL of PBS and subjected to transmission electron microscopy (TEM) (× 200) (JEOL, Akishima, Japan), cell co-culture, RNA extraction with Trizol reagent or protein detection with RIPA lysis buffer [30].

### Statistical analysis

Each experiment was performed in triplicate at least and all experimental data were manifested as the mean ± standard deviation (SD). Pearson’s correlation coefficient analysis was used to assess the linear correlations. The differences between groups were analyzed by the one-way analysis of variance (ANOVA) with Tukey’s test or Student’s *t* test as appropriate. The statistical analyses were performed using Graphpad Prism 7 software (GraphPad Inc., La Jolla, CA, USA). *P* < 0.05 suggested statistically significant.

## Results

### HG suppresses trophoblast cell viability, migration, invasion, and induces apoptosis in vitro

**Fig. 1:**
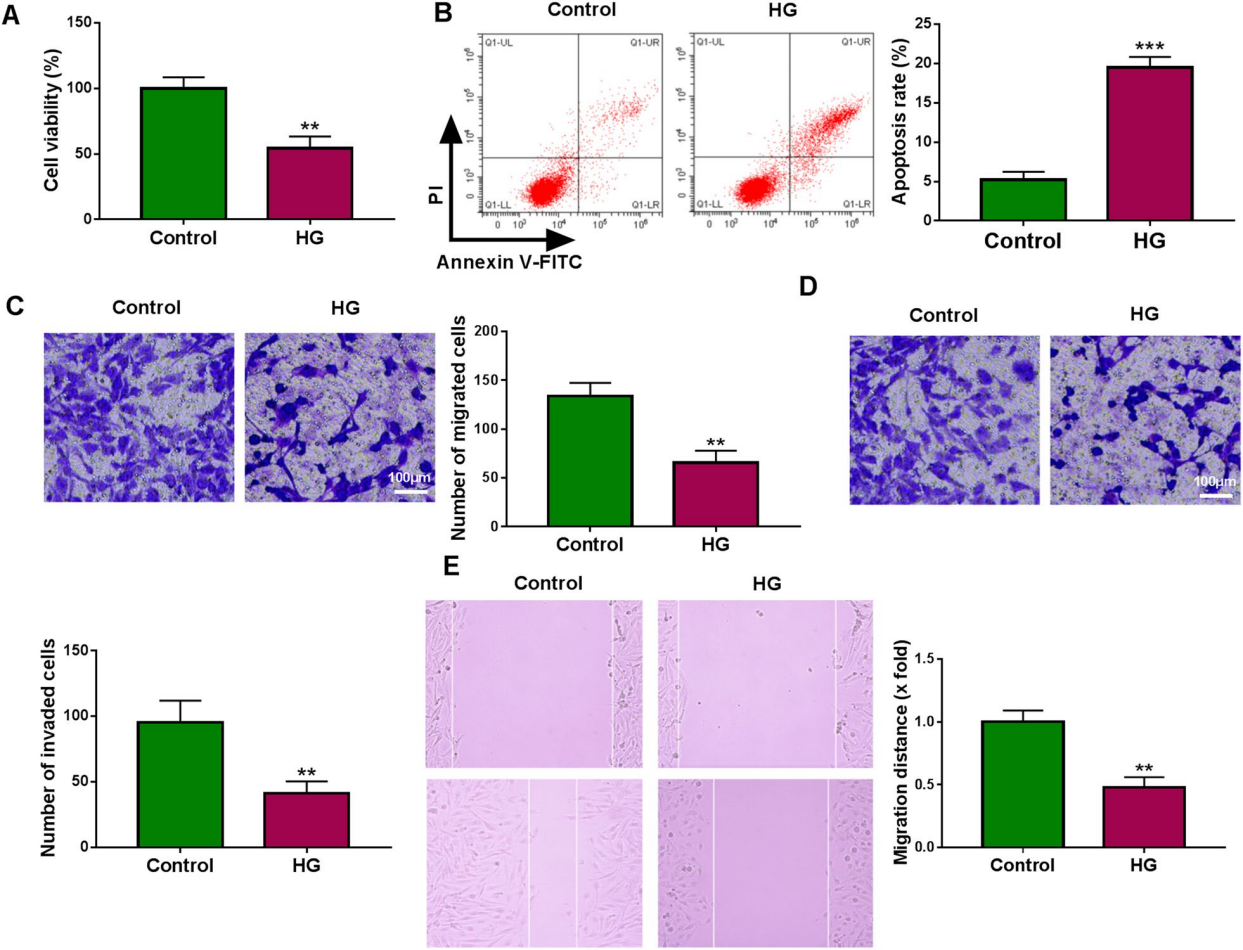
HG suppresses trophoblast cell viability, migration, invasion, and induces apoptosis in vitro. **A**–**E** HTR8/SVneo cells were treated with HG or normal glucose (control) for 48 h. **A** CCK-8 assay for cell viability. **B** Flow cytometric analysis of cell apoptosis. **C**, **D** Transwell assay of cell migration and invasion. **E** Wound healing assay for cell migration. **P < 0.01, ***P < 0.001

The effects of HG on the functions of trophoblast cells were firstly investigated. By contrast with cells with normal glucose (control), HG suppressed the viability (Fig. 1A) but induced apoptosis (Fig. 1B) in HTR8/SVneo cells. Then transwell assay showed the migration and invasion of HTR8/SVneo cells were repressed by the treatment of HG (Fig. 1C, D). Moreover, wound healing assay further suggested that HG led to a suppression of HTR8/SVneo cell migration (Fig. 1E). Therefore, we concluded that HG restrained cell growth and transfer processes in trophoblast cells.

### Circ-PNPT1 is highly expressed in placental tissues of GDM and HG-stimulated trophoblast cells

**Fig. 2:**
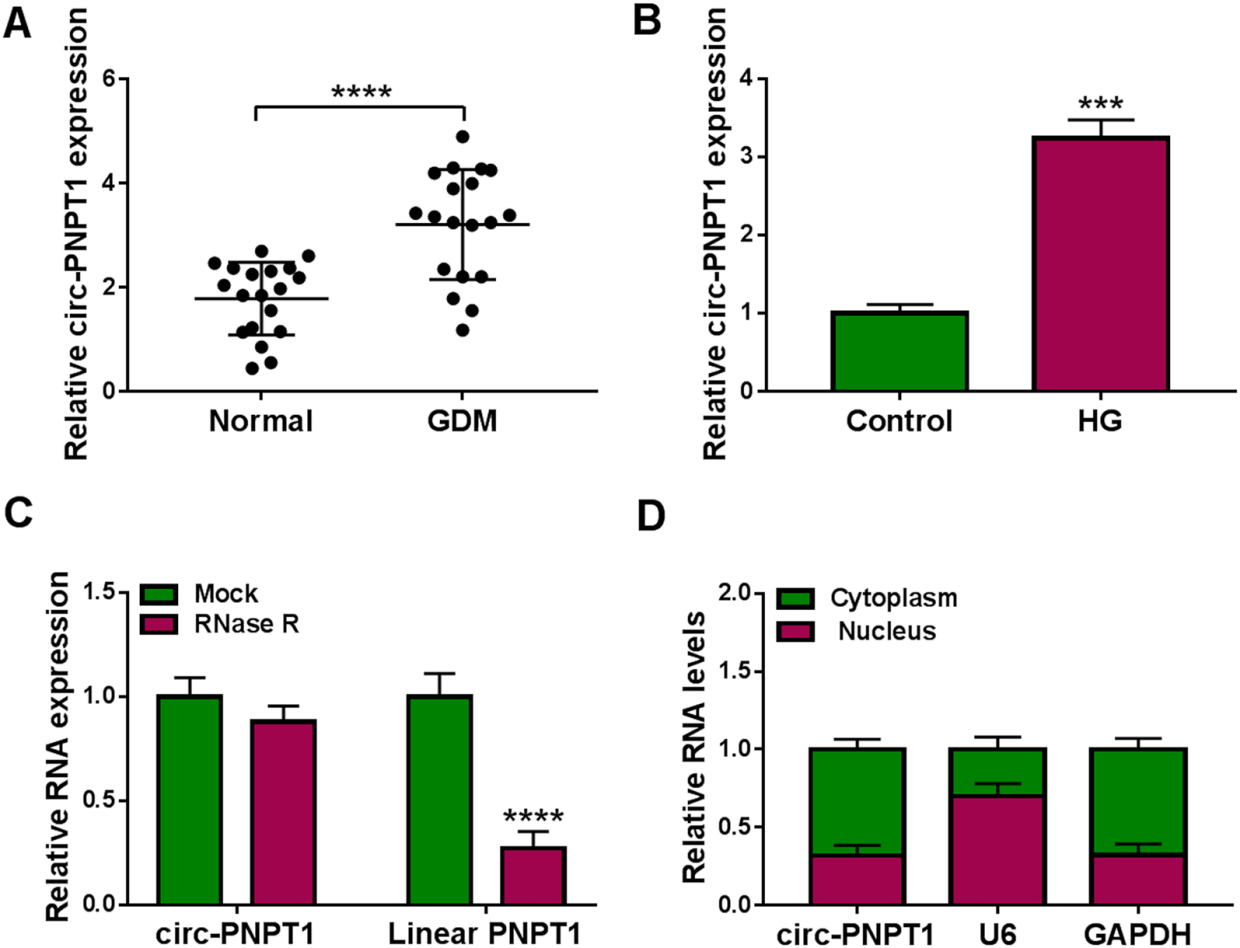
Circ-PNPT1 is highly expressed in placental tissues of GDM and HG-stimulated trophoblast cells. **A** qRT-PCR analysis of circ-PNPT1 expression in placental tissues with GDM or not. **B** qRT-PCR analysis of circ-PNPT1 expression in HTR8/SVneo cells treated with HG or normal glucose (control) for 48 h. **C** qRT-PCR analysis of circ-PNPT1 and linear PNPT1 mRNA expression in HTR8/SVneo cells after RNase R treatment. **D** qRT-PCR analysis of the levels of circ-PNPT1, GAPDH, and U6 in purified HTR8/SVneo nuclear and cytoplasmic fractions. ***P < 0.001, ****P < 0.0001

To elucidate the function of circ-PNPT1 in GDM, the expression profile of circ-PNPT1 was firstly detected. As shown in Fig. 2A, the expression of circ-PNPT1 was significantly elevated in placental tissues of GDM compared with the normal pregnancy placental tissues. Similarly, a significant elevation of circ-PNPT1 level in HG-induced HTR8/SVneo cells was observed (Fig. 2B). Thereafter, RNase R digestion was performed to investigate the circular characteristics of circ-PNPT1, results showed that circ-PNPT1 was resistant to RNase R digestion, while the linear form of PNPT1 decreased sharply under the RNase R treatment (Fig. 2C). Besides that, it was also discovered that circ-PNPT1 was predominantly distributed in cytoplasmic fraction of HTR8/SVneo cells (Fig. 2D). All these data suggested that circ-PNPT1 was an abundant, circular and stable transcript, and its elevation might be associated with GDM process.

### Circ-PNPT1 knockdown protects trophoblast cell from HG-induced cell growth and transfer processes inhibition

**Fig. 3:**
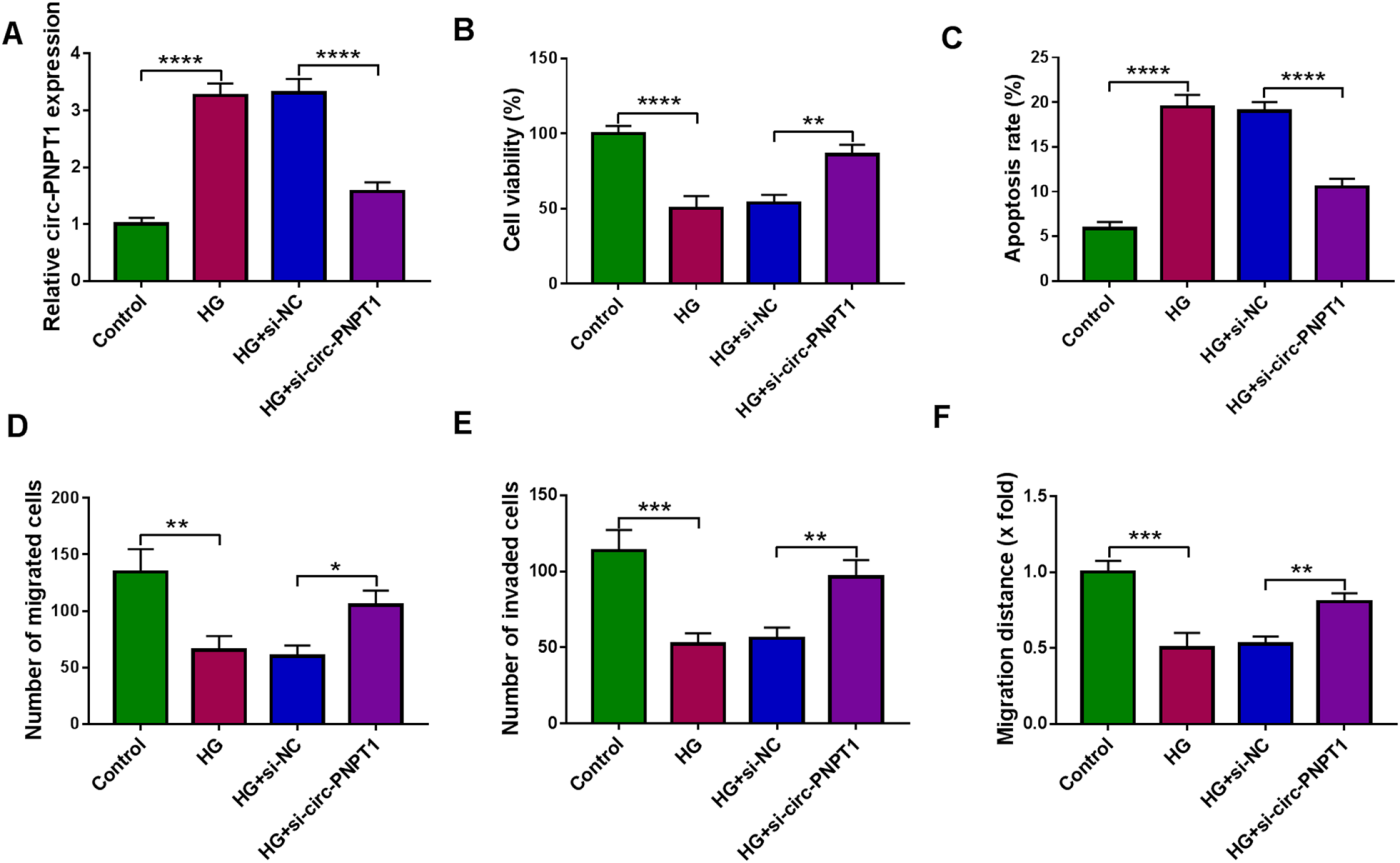
Circ-PNPT1 knockdown protects trophoblast cell from HG-induced cell growth and transfer processes inhibition. **A**–**F** HTR8/SVneo cells were transfected with si-circ-PNPT1 or si-NC and then subjected to HG stimulation. **A** qRT-PCR analysis of circ-PNPT1 expression in cells. **B** Cell viability analysis using CCK-8 assay. **C** Cell apoptosis analysis with flow cytometry. **D**, **E** Cell migration and invasion abilities analysis using transwell assay. **F** Wound healing assay for cell migration. *P < 0.05, **P < 0.01, ***P < 0.001, ****P < 0.0001

Given that circ-PNPT1 was highly expressed in HG-stimulated trophoblast cells, we hypothesized that HG might regulate circ-PNPT1 expression and secretion in trophoblast cells, and in turn, circ-PNPT1, as communication factors, modulated the function of trophoblast cells during GDM. To confirm this hypothesis, siRNA targeting circ-PNPT1 was designed and qRT-PCR analysis showed that the introduction of si-circ-PNPT1 significantly reduced circ-PNPT1 expression compared with si-NC transfection in HTR8/SVneo cells. Then transfected HTR8/SVneo cells were subjected to HG stimulation (Additional file 1: Fig. S1). As expected, the transfection of si-circ-PNPT1 reduced HG-induced elevation of circ-PNPT1 level in HTR8/SVneo cells (Fig. 3A). After the cell models were successfully constructed, cell viability and apoptosis were detected. The data of CCK-8 and flow cytometry manifested that knockdown of circ-PNPT1 notably reversed HG-induced viability arrest (Fig. 3B) and apoptosis (Fig. 3C) in HTR8/SVneo cells. Meanwhile, transwell and wound healing assays confirmed that circ-PNPT1 down-regulation reduced HG-evoked suppression of HTR8/SVneo cell migration and invasion (Fig. 3D–F). Taken together, knockdown of circ-PNPT1 abolished HG-induced trophoblast cell dysfunction, thus impeding GDM process.

### MiR-889-3p is a target of circ-PNPT1

**Fig. 4:**
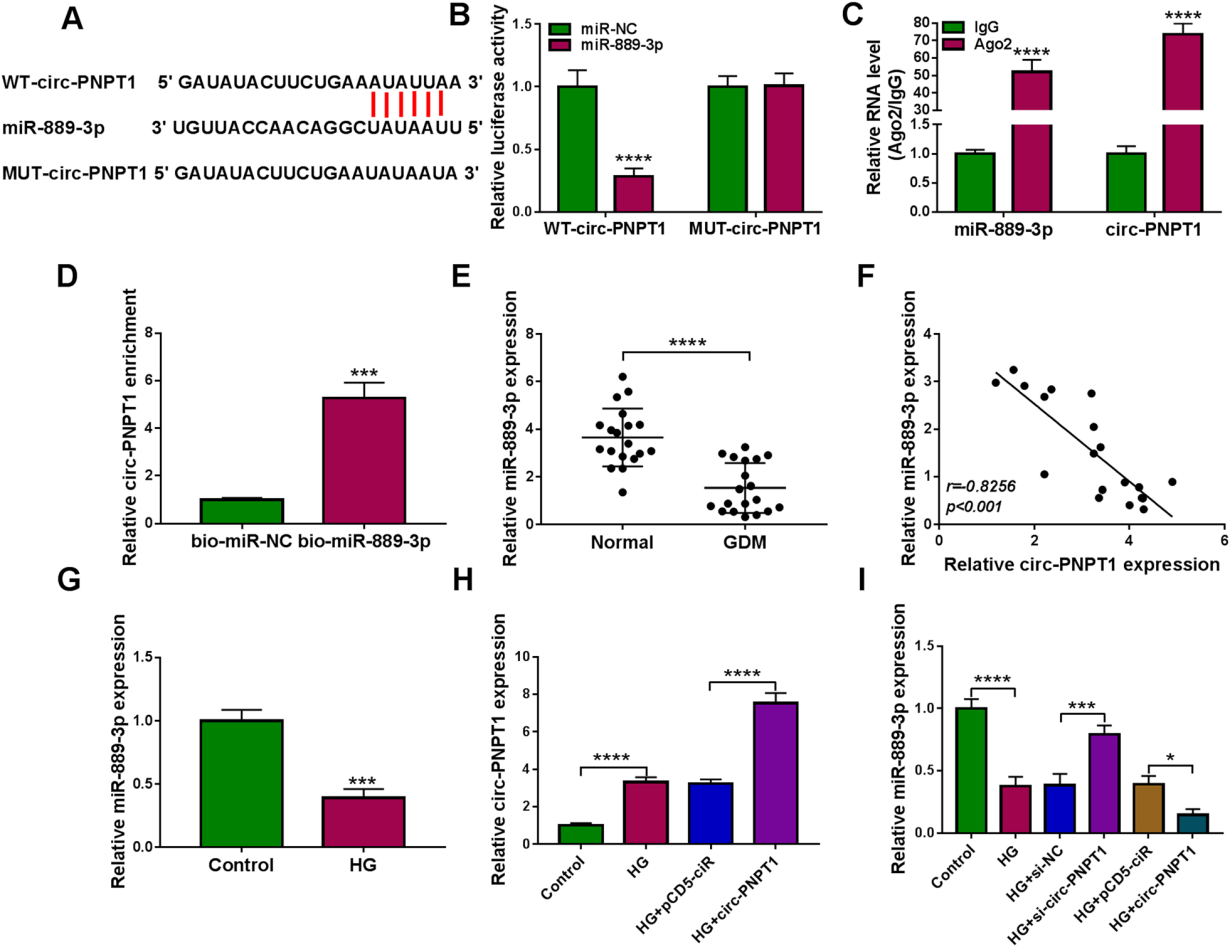
MiR-889-3p is a target of circ-PNPT1.** A** The potential binding site between circ-PNPT1 and miR-889-3p. **B**–**D** The interaction between miR-889-3p and circ-PNPT1 was confirmed using dual luciferase reporter assay, RIP assay and pull-down assay. **E** qRT-PCR analysis of miR-889-3p expression in placental tissues with GDM or not. **F** The correlation between miR-889-3p and circ-PNPT1 expression in placental tissues was analyzed by Pearson analysis. **G** qRT-PCR analysis of miR-889-3p expression in HTR8/SVneo cells treated with HG or normal glucose (control) for 48 h. **H** qRT-PCR analysis of circ-PNPT1 level in HTR8/SVneo cells transfected with pCD5-ciR or circ-PNPT1 under HG treatment. **I** qRT-PCR analysis of miR-889-3p expression in HTR8/SVneo cells transfected with pCD5-ciR, circ-PNPT1, si-NC, or si-circ-PNPT1 in the presence of HG. *P < 0.05, ***P < 0.001, ****P < 0.0001

Through searching the circinteractome database, a potential binding site between circ-PNPT1 and miR-889-3p was found (Fig. 4A). Then we further confirmed whether circ-PNPT1 could absorb miR-889-3p. Results of dual luciferase reporter assay showed that miR-889-3p overexpression markedly reduced the luciferase activity of WT-circ-PNPT1 reporter but not the MUT-circ-PNPT1 reporter relative to the control group in HTR8/SVneo cells (Fig. 4B). RIP assay implied that circ-PNPT1 and miR-889-3p were enriched in Ago2-containing microribonucleoproteins compared to control IgG (Fig. 4C). Furthermore, we also found that circ-PNPT1 was overtly pulled down by bio-miR-889-3p probe in HTR8/SVneo cells (Fig. 4D). All these data confirmed that miR-889-3p was a target of circ-PNPT1.

Next, the expression profile of miR-889-3p was investigated. We discovered that miR-889-3p expression was decreased in placental tissues of GDM (Fig. 4E), which was negatively correlated with circ-PNPT1 expression (Fig. 4F). Also, miR-889-3p expression was low in HG-stimulated HTR8/SVneo cells (Fig. 4G). Besides that, after confirming the transfection of circ-PNPT1 plasmid using qRT-PCR in HG-mediated HTR8/SVneo cells (Fig. 4H), it was proved that circ-PNPT1 down-regulation relieved HG-induced reduction of miR-889-3p expression, while circ-PNPT1 overexpression showed opposite effects (Fig. 4I). Altogether, circ-PNPT1 directly targeted miR-889-3p and negatively regulated its expression.

### MiR-889-3p inhibition attenuates the effects of circ-PNPT1 knockdown on HG-induced trophoblast cells

**Fig. 5:**
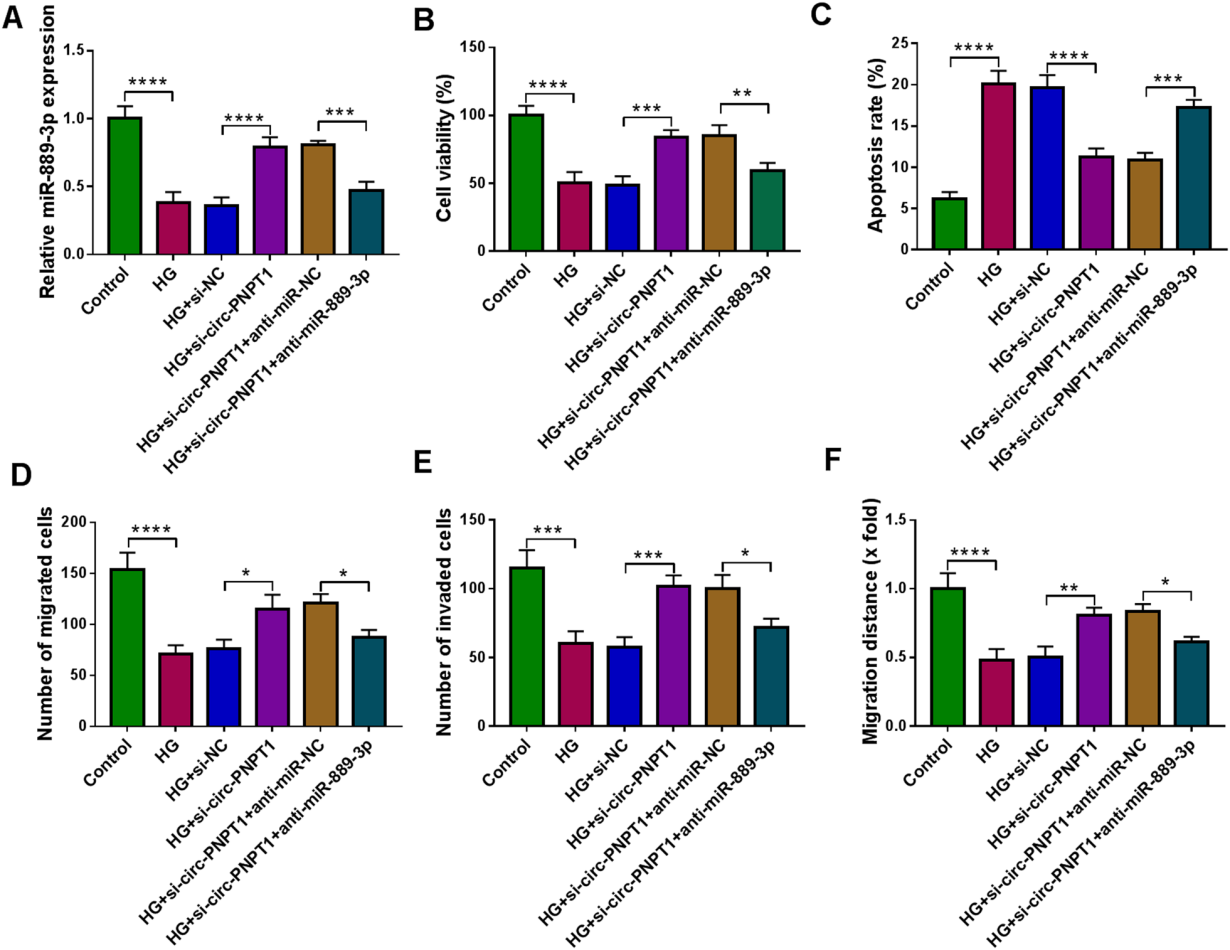
MiR-889-3p inhibition attenuates the effects of circ-PNPT1 knockdown on HG-induced trophoblast cells. **A**–**F** HTR8/SVneo cells were co-transfected with si-circ-PNPT1 and/or anti-miR-889-3p and then subjected to HG stimulation. **A** qRT-PCR analysis of miR-889-3p expression in cells. **B** Cell viability analysis with CCK-8 assay. **C** Cell apoptosis analysis with flow cytometry. **D**, **E** Cell migration and invasion abilities analysis using transwell assay. **F** Wound healing assay for cell migration. *P < 0.05, **P < 0.01, ***P < 0.001, ****P < 0.0001

The data mentioned above indicated that miR-889-3p was a downstream molecule of circ-PNPT1, we then explored whether circ-PNPT1/miR-889-3p axis was responsible for GDM process. Circ-PNPT1 siRNA and miR-889-3p inhibitor were co-transfected into HTR8/SVneo cells. The transfection efficiency was validated by qRT-PCR (Fig. 5A). After that, it was demonstrated that circ-PNPT1 knockdown induced cell viability (Fig. 5B), migration and invasion promotion (Fig. 5D–F) and apoptosis arrest (Fig. 4C) in HG-mediated HTR8/SVneo cells, while these effects were partially abolished by the co-transfection of miR-889-3p inhibitor (Fig. 5B–F). These studies indicated that circ-PNPT1/miR-889-3p axis was engaged in GDM process.

### PAK1 is a target of miR-889-3p

**Fig. 6:**
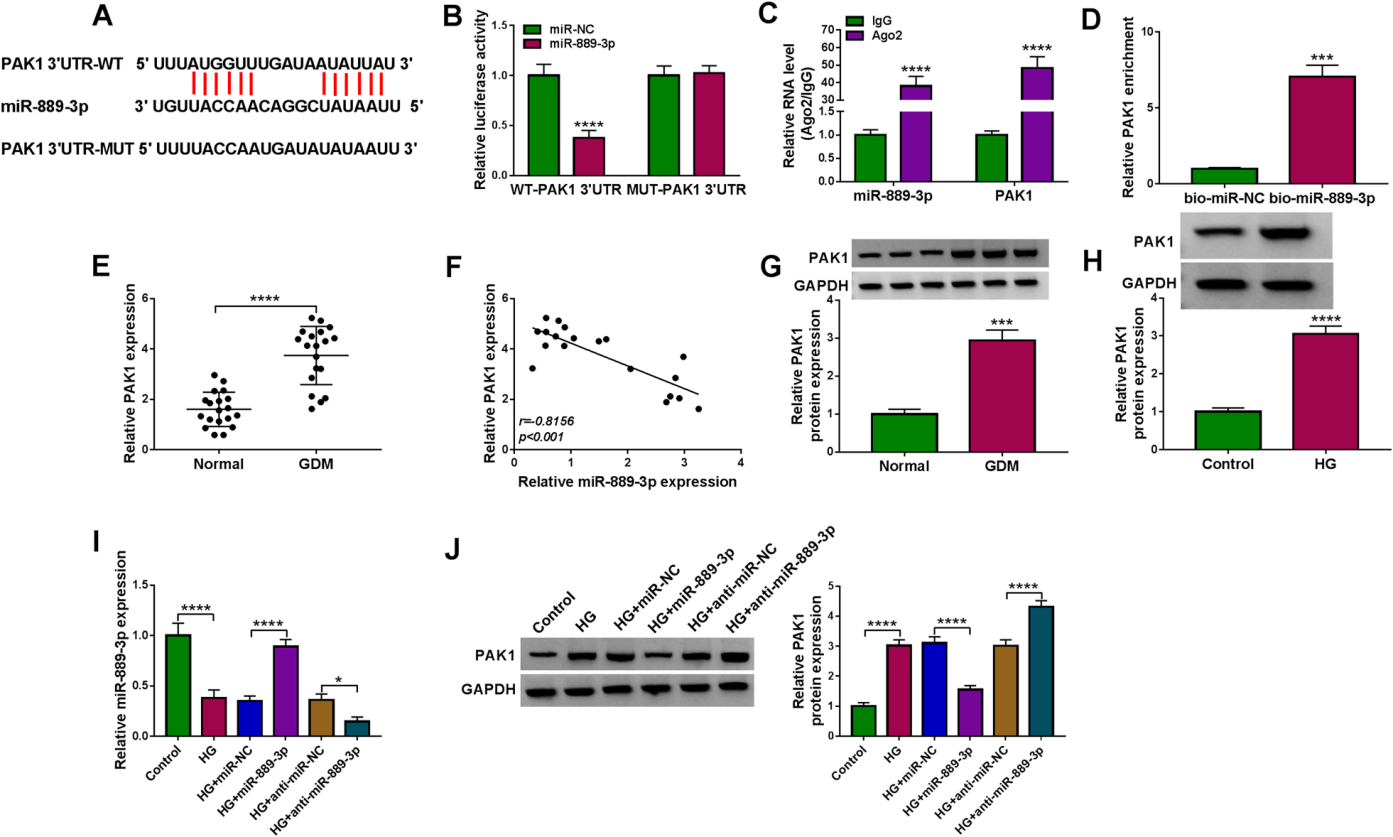
PAK1 is a target of miR-889-3p. **A** The putative miR-889-3p binding sites on the 3′UTR of PAK1. **B**–**D** The interaction between miR-889-3p and PAK1 was confirmed using dual luciferase reporter assay, RIP assay and pull-down assay. **E** qRT-PCR analysis of PAK1 mRNA expression in placental tissues with GDM or not. **F** The correlation between miR-889-3p and PAK1 expression in placental tissues was analyzed by Pearson analysis. **G** Western blot analysis of PAK1 protein expression in placental tissues with GDM or not. **H** Western blot analysis of PAK1 protein expression in HTR8/SVneo cells treated with HG or normal glucose (control) for 48 h. **I**, **J** HTR8/SVneo cells were transfected with miR-NC, miR-889-3p, anti-miR-NC, or anti-miR-889-3p and then treated with HG. **I** qRT-PCR analysis of miR-889-3p level in HTR8/SVneo. **J** Western blot analysis of PAK1 protein in HTR8/SVneo cells. *P < 0.05, ***P < 0.001, ****P < 0.0001

The downstream targets of miR-889-3p were further searched. Based on the prediction of Starbase2.0 database, we found that PAK1 might be a target gene of miR-889-3p. The putative miR-889-3p binding sites on the 3′UTR of PAK1 were showed in Fig. 6A. After that, dual-luciferase reporter assay suggested that the luciferase activity of WT-PAK1 3′UTR could be notably reduced by miR-889-3p up-regulation in HTR8/SVneo cells, while no changes were observed in MUT-PAK1 3′UTR reporter (Fig. 6B). RIP assay revealed that miR-889-3p and PAK1 was specifically pulled down by Ago2 antibody relative to the control IgG antibody in HTR8/SVneo cells (Fig. 6C). Moreover, pull-down assay indicated that PAK1 was significantly more abundant in bio-miR-889-3p group compared with the control (Fig. 6D). All these results confirmed the direct interaction of miR-889-3p and PAK1.

Next, the expression pattern of PAK1 in GDM was detected. PAK1 was found to be highly expressed in placental tissues of GDM (Fig. 6E, G), which was negatively correlated with miR-889-3p expression (Fig. 6F). Similarly, its expression was also increased in HG-stimulated HTR8/SVneo cells (Fig. 6H). After confirming the transfection efficiencies of miR-889-3p mimic and inhibitor using qRT-PCR (Fig. 6I), it was discovered that miR-889-3p overexpression reduced HG-induced elevation of PAK1 level, while its down-regulation enhanced HG-induced elevation of PAK1 level (Fig. 6J), suggesting that miR-889-3p targetedly suppressed PAK1 expression.

### MiR-889-3p protects trophoblast cell from HG-induced cell growth and transfer processes inhibition

**Fig. 7:**
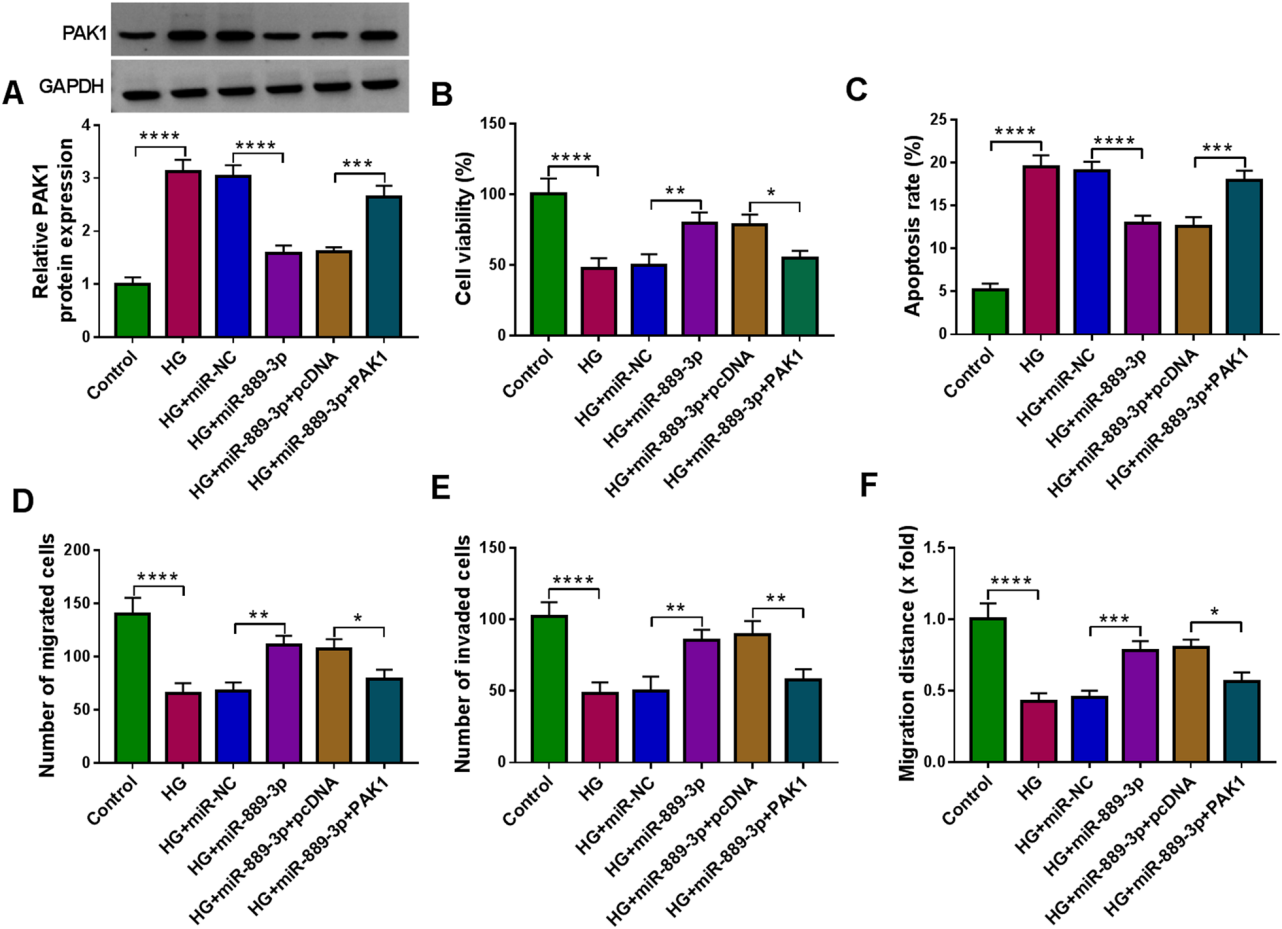
MiR-889-3p protects trophoblast cell from HG-induced cell growth and transfer processes inhibition. **A**–**F** HTR8/SVneo cells were transfected with miR-NC, miR-889-3p, miR-889-3p + pcDNA, or miR-889-3p + PAK1, followed by treatment with HG. **A** Western blot analysis of PAK1 protein level in HTR8/SVneo cells. **B** CCK-8 assay for cell viability. **C** Flow cytometric analysis of cell apoptosis. **D**, **E** Transwell assay of cell migration and invasion. **F** Wound healing assay for cell migration. *P < 0.05, **P < 0.01, ***P < 0.001, ****P < 0.0001

We then illustrated whether miR-889-3p/PAK1 axis was engaged in GDM process. HTR8/SVneo cells were transfected with miR-NC, miR-889-3p, miR-889-3p + pcDNA, or miR-889-3p + PAK1, followed by treatment with HG. Western blot analysis suggested that PAK1 transfection rescued miR-889-3p-induced decrease of PAK1 level in HG-mediated HTR8/SVneo cells (Fig. 7A). Thereafter, rescue assay was conducted. We found that miR-889-3p overexpression reduced HG-induced cell viability arrest (Fig. 7B) and apoptosis (Fig. 7C), which were attenuated by PAK1 up-regulation. Furthermore, it was also demonstrated that PAK1 up-regulation impaired miR-889-3p re-expression-triggered migration and invasion in HG-stimulated HTR8/SVneo cells (Fig. 7D–F). Collectively, we confirmed that miR-889-3p abolished HG-induced trophoblast cell dysfunction via targeting PAK1, thus hindering GDM process.

### Circ-PNPT1 leads to trophoblast cell dysfunction via miR-889-3p/PAK1 axis

**Fig. 8:**
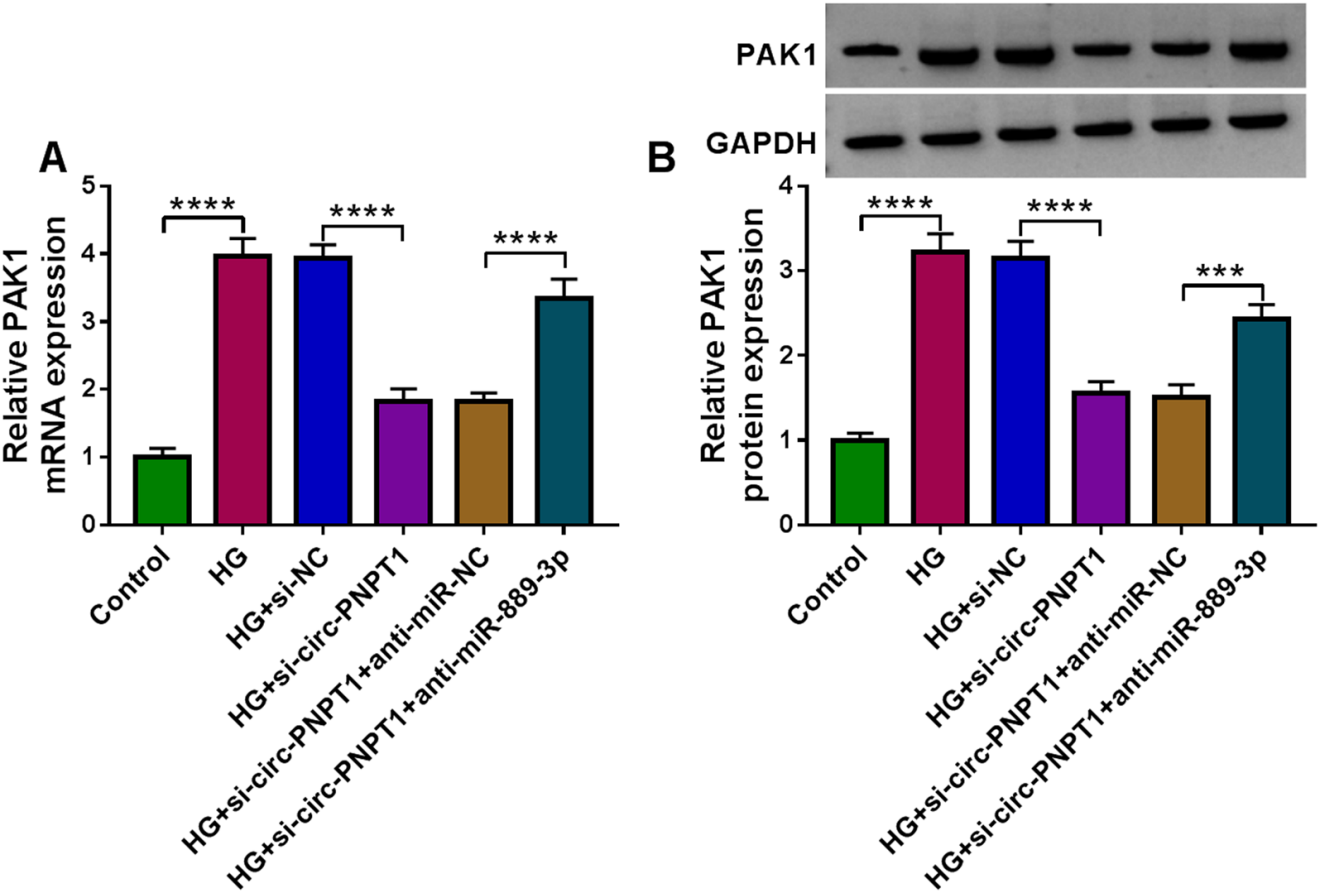
Circ-PNPT1 leads to trophoblast cell dysfunction via miR-889-3p/PAK1 axis. HTR8/SVneo cells were transfected with si-NC, si-circ-PNPT1, si-circ-PNPT1 + anti-miR-NC, or si-circ-PNPT1 + anti-miR-889-3p, followed by treatment with HG. **A**, **B** qRT-PCR and Western blot analysis of PAK1 level in HTR8/SVneo cells. ***P < 0.001, ****P < 0.0001

Considering the aforementioned results, we then studied whether circ-PNPT1 could regulate PAK1 via miR-889-3p. As presented in Fig. 8A, B, we discovered that circ-PNPT1 knockdown led to a reduction of PAK1 expression, which was reverted by miR-889-3p down-regulation in HG-induced HTR8/SVneo cells. Altogether, a circ-PNPT1/miR-889-3p/PAK1 axis was identified.

### Extracellular circ-PNPT1 is packaged into exosomes and can be internalized by trophoblast cells

**Fig. 9:**
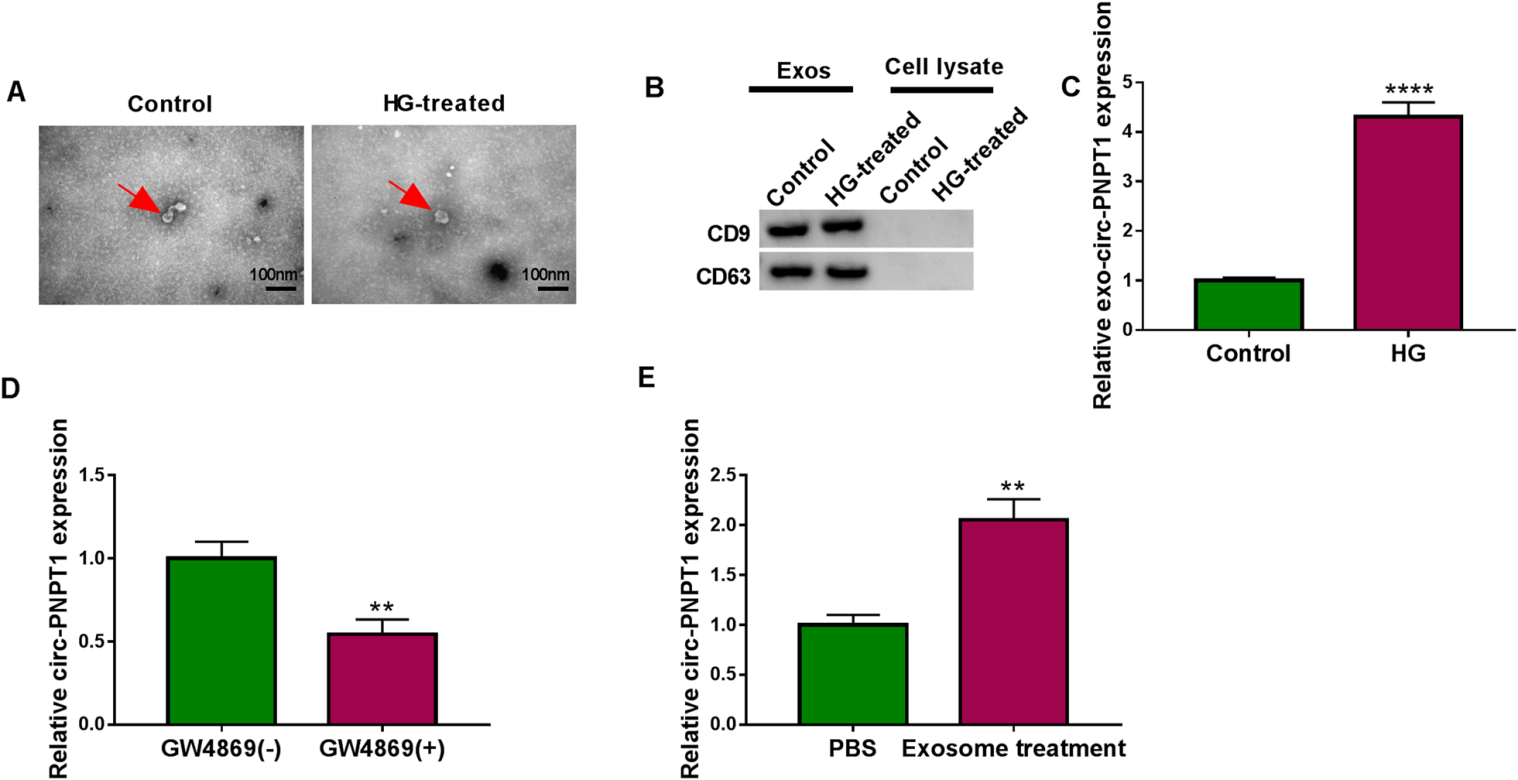
Extracellular circ-PNPT1 is packaged into exosomes and can be internalized by trophoblast cells. **A** Representative TEM images of exosomes from HTR8/SVneo cells treated with HG or not. **B** Western blot analysis of CD9 and CD63 in exosomes and cell lysate. **C** qRT-PCR analysis of circ-PNPT1 expression in exosomes isolated from HTR8/SVneo cells treated with HG or not. **D** qRT-PCR analysis of circ-PNPT1 expression in the cell media of HG-induced HTR8/SVneo cells which were co-cultured with or without GW4869. **E** qRT-PCR analysis of circ-PNPT1 expression in HTR8/SVneo cells co-cultured with exosomes carrying circ-PNPT1 or PBS. **P < 0.01, ****P < 0.0001

According to recent studies that exosomes can be actively secreted by most cell types, and exosome-mediated transfer of circRNAs can mediate signals between cells [31–33]. Therefore, we explored the existing pattern of extracellular circ-PNPT1. The exosomes in the culture media of treated or untreated HTR8/SVneo cells were isolated. TEM analysis confirmed the presence of translucent, cup-shaped vesicles (Fig. 9A), the detection of surface hallmarks (CD63 and CD9) by western blot further verified that the isolated particles were exosomes (Fig. 9B). After that, we found that circ-PNPT1 expression was higher in exosomes isolated from HG-induced HTR8/SVneo cells than that from untreated HTR8/SVneo cells (Fig. 9C). Moreover, HG-induced HTR8/SVneo cells were co-cultured with or without GW4869, an inhibitor of exosome generation, we found the level of circ-PNPT1 expression in cell media were blocked by GW4869 (Fig. 9D), suggesting that extracellular circ-PNPT1 was packaged into exosomes. Besides that, untreated HTR8/SVneo cells were co-cultured with exosomes or PBS, and qRT-PCR analysis showed circ-PNPT1 level was significantly increased in HTR8/SVneo cells treated with exosomes compare with PBS treatment (Fig. 9E), which indicated exosomal circ-PNPT1 served as a mediator in intercellular communication between cells.

## Discussion

The placenta is a temporary organ, acting as the link between the mother and fetus, which supports intrauterine life, besides that, it has nutritional, endocrine and immunologic functions to maintain fetal development [7, 34]. Recently, the placenta is increasingly appreciated to be a target organ of GDM, maternal hyperglycemia, as observed in GDM, affects placental structure, which may lead to functional changes in this organ, facilitating fetus malformation and abortion [34, 35]. The normal function of trophoblast cells is important for placenta development, cell migration, invasion and growth inhibition may be involved in the maldevelopment of placenta [25]. CircRNAs are covalently closed, single-stranded transcripts and have been identified to involve in the dysfunction of trophoblastic cells. For instance, hsa_circ_0000848 mediated cell invasion and migration promotion and apoptosis inhibition in placental trophoblast cells via regulating hsa-miR-6768-5p [36]. Zhou’s team suggested that circZDHHC20 up-regulation impaired trophoblast cell viability, migration and invasion through elevating GRHL2 expression via absorbing miR-144 [27].

In this study, circ-PNPT1 was found to be highly expressed in the placental tissues of GDM and HG-induced trophoblast cells. It was found that HG treatment induced apoptosis and suppressed the viability, invasion and migration in trophoblast cells. Importantly, when we down-regulated the expression of circ-PNPT1 in HG-induced trophoblast cells, decrease of circ-PNPT1 significantly reversed HG-mediated dysfunction of trophoblast cells. Thus, we concluded that knockdown of circ-PNPT1 attenuated HG-induced trophoblast cell dysfunction, suggesting that silencing of circ-PNPT1 might have a protective role in the progression of GDM.

It has been revealed that circRNAs can act as “miRNA sponge”, offsetting miRNA-mediated degradation of mRNAs, and form a competing endogenous RNA (ceRNA) network with miRNA downstream mRNA to modulate diverse biological processes [37–39]. Thus, the specific regulatory network underlying circ-PNPT1 was then investigated. The underlying mechanism suggested that serving as a ceRNA, circ-PNPT1 directly targeted miR-889-3p to positively up-regulate PAK1 expression. In this study, miR-889-3p was found to be decreased, while PAK1 expression was increased in the placental tissues of GDM and HG-induced trophoblast cells. Functionally, re-expression of miR-889-3p abolished HG-induced growth and transfer processes inhibition by directly targeting PAK1, moreover, miR-889-3p suppression reduced the inhibitory effects of circ-PNPT1 knockdown on HG stimulated trophoblast cell dysfunction. Taken together, a circ-PNPT1/miR-889-3p/PAK1 regulatory network in trophoblast cell dysfunction was identified.

Interestingly, this study also found circ-PNPT1 expression was higher in the exosomes isolated from HG-induced trophoblast cells and confirmed that it was mainly located in exosomes. Importantly, it was also proved that isolated exosomes carrying high circ-PNPT1 could be transferred into normal trophoblast cells. Exosomes are small spherical packages that can be released by most cell types, they are mediators of intercellular communication through the transfer of their contents, which can affect cell biological behaviors, thus involving in the physiological and pathological conditions of human diseases [19, 23]. Exosomes are stable presence in the blood and other bodily fluids due to their phospholipid bilayer and endogenous origin [40, 41], besides, exosomes can avoid immune recognition and clearance [42], thus, there is growing interest in using exosomes as in vivo transporters for circRNAs [19]. Importantly, it has been uncovered that exosomes loaded with therapeutic RNAs can be manufactured in bulk by exosome producing cells in vitro thus enabling personalized treatment [43, 44]. Therefore, exosomal circ-PNPT1 might be an ideal biomarker for therapeutic intervention for GDM.

**Fig. 10:**
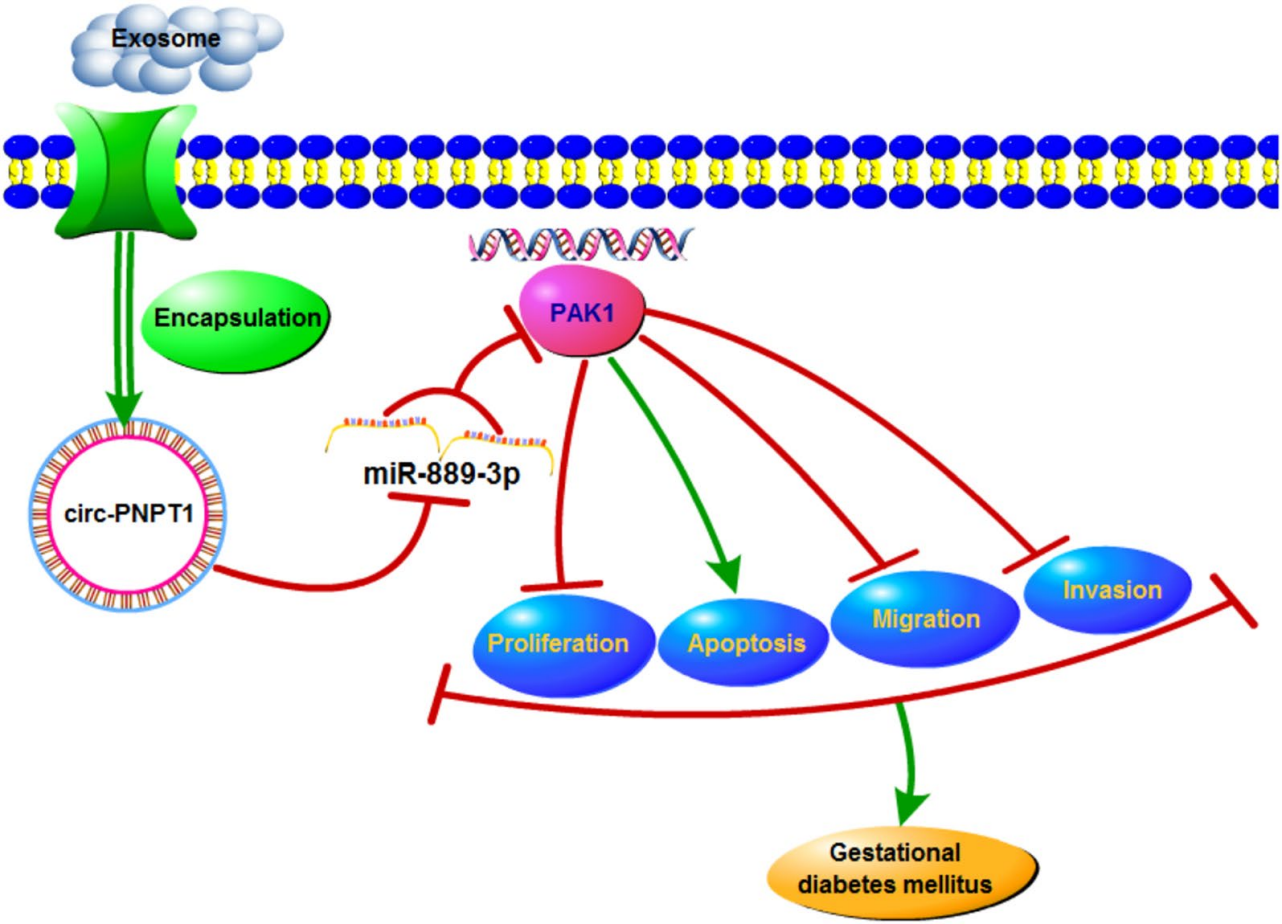
Graphical abstract of how circ-PNPT1 involves in trophoblast cell dysfunction in GDM. Circ-PNPT1 secreted by exosomes sponges miR-889-3p to up-regulate PAK1 level to reduce trophoblast cell growth and transfer processes

In conclusion, this study demonstrated that circ-PNPT1 contributed to HG-induced suppression of trophoblast cell proliferation, migration and invasion via the miR-889-3p/PAK1 axis (Fig. 10), suggesting a novel insight into the pathogenesis of GDM.

## Supplementary Information

**Additional file 1: Fig. S1. **The effects of circ-PNPT1 siRNA. qRT-PCR analysis of circ-PNPT1 expression in HTR8/SVneo cells transfected with si-circ-PNPT1 or si-NC. ***P < 0.001.

## Data Availability

Not applicable.
