# Supplementary figures and images for: Circ-PNPT1 contributes to gestational diabetes mellitus (GDM) by regulating the function of trophoblast cells through miR-889-3p/PAK1 axis
Source: Diabetol Metab Syndr. 2021 Jun 1;13:58. doi: 10.1186/s13098-021-00678-9 (PMC8171017; doi:10.1186/s13098-021-00678-9)

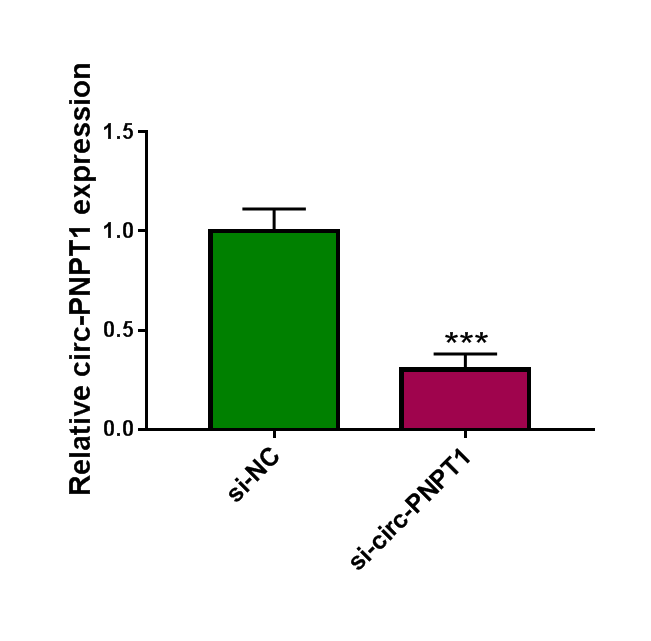

Supplement: Supplementary file 1 — Additional file 1: Fig. S1. The effects of circ-PNPT1 siRNA. qRT-PCR analysis of circ-PNPT1 expression in HTR8/SVneo cells transfected with si-circ-PNPT1 or si-NC. ***P < 0.001. [file 13098_2021_678_MOESM1_ESM.tif]
